# Encapsulating Proton Inside C_60_ Fullerene: A Density Functional Theory Study on the Electronic Properties of Cationic X^+^@C_60_ (X^+^ = H^+^, H_3_O^+^ and NH_4_^+^)

**DOI:** 10.3390/ijms252212014

**Published:** 2024-11-08

**Authors:** Lei Zhao, Bo Wang

**Affiliations:** School of Science, Northeast Electric Power University, Jilin 131200, China; 20172736@neepu.edu.cn

**Keywords:** proton, encapsulated fullerene, electronic structure, density functional theory, ionic species

## Abstract

Confining protons into an enclosed carbon cage is expected to give rise to unique electronic properties for both the inner proton and the outer cage. In this work, we systematically investigated the geometric and electronic structures of cationic X^+^@C_60_ (X^+^ = H^+^, H_3_O^+^, and NH_4_^+^), and their corresponding neutral species (X = H_2_O, NH_3_), by quantum chemical density functional theory calculations. We show that C_60_ can trap H_2_O, NH_3_, H_3_O^+^ and NH_4_^+^ at the cage center and only slightly influence their geometries. The single proton clings to the inner wall of C_60_, forming a C-H chemical bond. The encapsulated neutral species almost do not change the electronic structure of the C_60_, while the internal cations have obvious effects. The charge transfer effect from the inner species to the C_60_ cage was found for all X@C_60_ (X = H_2_O, NH_3_) (about 0.0 e), X^+^@C_60_ (X^+^ = H_3_O^+^, NH_4_^+^) (about 0.5 e) and H^+^@C_60_ (about 1.0 e) systems. Encapsulating different forms of protons also regulates the fundamental physico-chemical properties of the hollow C_60_, such as the HOMO-LUMO gaps, infrared spectra, and electrostatic potential, etc., which are discussed in detail. These findings provide a theoretical insight into protons’ applications, especially in energy.

## 1. Introduction

Protons in aqueous systems play a crucial role in various physical, chemical and biological processes [1,2]. For instance, they are involved in fuel cell membranes [3], ATP synthesis [4], enzymatic reactions in proteins [5], and more. When protons are in confined systems, they exhibit unique electronic, magnetic and optical properties [6,7]. Fullerenes, with hollow inner spaces, can encapsulate a wide variety of species, including atoms, molecules or ions, forming endohedral fullerenes [8,9,10]. They offer a potential platform for studying the behavior of protons in a confined setting. The unique structure of fullerenes not only provides a confined space but also may interact with the protons in ways that could further enhance our understanding of protons’ behavior and their associated properties [11,12,13,14,15]. The inner space of C_60_, with a diameter of 3.53 Å, is suitable for entrapping a guest species [16,17]. It provides a remarkably simple model system through which to study the properties of species in confinement. As of now, a large number of experimental and theoretical methods have been used to research molecule endohedral fullerenes, such as H_2_O@C_60_ [17,18,19,20,21,22,23,24,25,26,27,28,29,30], HF@C_60_ [26,31,32], H_2_@C_60_ [33,34,35,36], etc. These endohedral fullerenes have been synthesized by means of the arc discharge method [37] and molecular surgical method [38,39]. Some theoretical research has been carried out on H^+^ and NH_3_ embedded into C_60_ [40,41,42]. The studies focused on the geometry parameter, interaction energy, charge transfer, and spectra.

The proton, as an elementary particle, differs from all other ions that are stable in aqueous solutions. When introduced into water, a proton does not exist independently but rapidly combines with neighboring water molecules to form a hydronium cation (H_3_O^+^) [43]. Protons show the same behavior in ammonia, forming ammonium ion (NH_4_^+^) [44]. Moreover, the hydration of H_3_O^+^ and NH_4_^+^ plays an important role in the physical and natural sciences [45,46]. This is due to hydrogen bonding network formed in aqueous acid–base chemistry. Meanwhile, H_3_O^+^ batteries are considered one of the most promising energy technologies as a next-generation power source, thanks to their cost-effectiveness and sustainability [47]. NH_4_^+^ and other species react to form the ammonium salt complex species in the natural environment [48]. However, little is known about encapsulation of H_3_O^+^ and NH_4_^+^ inside C_60_. Trapping H_3_O^+^ and NH_4_^+^ in a confined environment provides a unique opportunity to understand their novel properties. Hence, an in-depth study of the geometric and electronic structures of the cationic X^+^@C_60_ (X^+^ = H^+^, H_3_O^+^, and NH_4_^+^), as well as their corresponding neutral species (X = H_2_O, NH_3_), would certainly be a worthwhile exercise.

In this work, we present the results of first-principles computations to investigate the geometric and electronic structures of cationic X^+^@C_60_ (X^+^ = H^+^, H_3_O^+^, NH_4_^+^), as well as their corresponding neutral species (X = H_2_O, NH_3_). This paper is organized as follows. First, the computational methodology is described. Then, we present results for the systems, including optimized geometry parameters, interaction energy, charge populations, electron density difference, HOMO-LUMO gaps, infrared spectra, electrostatic potentials, and hydration free energies. Finally, important results are summarized in the conclusions section, which may be useful for furthering sustainable and efficient energy sources.

## 2. Results

**Figure 1:**
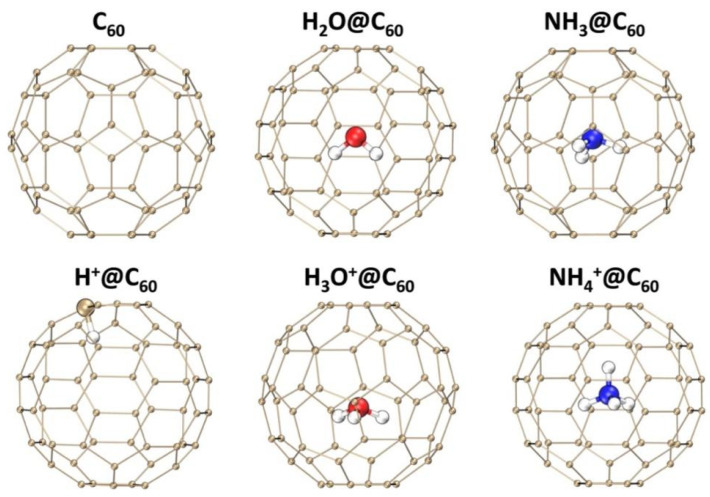
Structures of C_60_, X^+^@C_60_ (X^+^ = H^+^, H_3_O^+^, and NH_4_^+^) and X@C_60_ (X = H_2_O, NH_3_) at the B3LYP-D3(BJ)/6-31G(d, p) level of theory. C, H, O, and N atoms are indicated by orange, white, red, and blue spheres, respectively.

Figure 1 presents the optimized geometries of C_60_, X^+^@C_60_ (X^+^ = H^+^, H_3_O^+^, NH_4_^+^), and X@C_60_ (X = H_2_O, NH_3_) at B3LYP-D3(BJ)/6-31G(d, p) at the theoretical level (for coordinates for optimized molecular structure, see Supplementary Materials). The optimized geometry parameters of isolated and confined species are compared to investigate the interaction between the C_60_ cage and the confined species. The optimized geometry parameters are shown in Table 1. For the neutral species X encapsulated inside C_60_, species X is located at the center of the cage. The O-H and N-H bond lengths decrease by 0.001 Å and 0.004 Å, respectively, compared to the free molecule. Similar negligible changes have been reported in the literature [7,41,49], indicating that the computational level used in this work is reliable. Additionally, our calculations also show that the interaction between X and C_60_ is minimal, with almost no perturbation of the C_60_ geometry. It should be noted that in H^+^@C_60_, the H^+^ is attached to C atom, indicating that the H^+^ directly interacts with the C atom of C_60_. The C-H bond length in H^+^@C_60_ is 1.130 Å, which is 3.5% longer than that (1.092) of free CH_4_ due to C-H bond weakening. The H_3_O^+^ encapsulated inside fullerene cage shows a slight deviation from the center. The comparison of the H_3_O^+^ encapsulated inside C_60_ and free H_3_O^+^ in the gas phase reveals that the H_3_O^+^ encapsulated inside C_60_ has a particular conformation with a longer O-H (0.994 Å) than that of a free H_3_O^+^ (0.982 Å), being about 1.2% larger than the O-H bond length, which is consistent with the results of reference [22,40]. The small increase in bond length shows that the attraction of H toward the cage causes the slight stretch. However, for NH_4_^+^ encapsulated inside the fullerene cage, NH_4_^+^ is located at the center of the cage. The computed N-H bond length of 1.027 Å for the NH_4_^+^@C_60_ is consistent with that of the free NH_4_^+^ molecule. This result indicates that the interaction between H_3_O^+^ and C_60_ is stronger than that between NH_4_^+^ and C_60_.

## 3. Discussion

**Figure 2:**
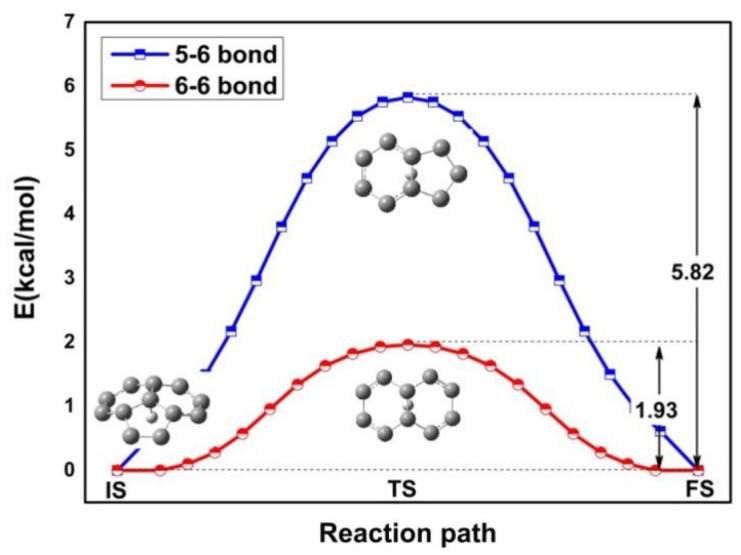
Energy profile for H diffusion from the atom to the 5-6 bond and 6-6 bond. The atomic geometries of the initial (IS), transition (TS), and final (FS) states are also given. The blue boxes and red circles indicate the 5-6 bond and 6-6 bond of the intrinsic reaction coordinate (IRC), respectively. The diffusion barrier is denoted by an arrow.

To generate the stable structure of H^+^ adsorbs, we previously considered proposed adsorption sites, atoms, bridges, and hollow sites. The optimization procedure of the C_60_ geometrical structure yielded the 5-6 bonds at the junctions between a five- and a six-membered ring and the 6-6 bonds at the junctions between a six- and a six-membered ring. The lowest-energy adsorption site is the atom site, where a C-H bond is formed. Figure 2 displays the calculated energy profile along pathway from the atom site to the bridge site. In the transition state, the H^+^ is located on the 5-6 bond and 6-6 bond. We carried out intrinsic reaction coordinate (IRC) calculations in two parts. The corresponding energy barriers are 5.82 kcal/mol and 1.93 kcal/mol, respectively. Our results show that the transition from the atom to the 6-6 bond is much easier than that to the 5-6 bond due to the low energy barrier, suggesting that the pathway proposed here is reasonable.

Energy decomposition analysis is a powerful method of assessing qualitative and quantitative interaction. Table 2 presents the contributions of electrostatic (∆E_elstat_), Pauli (∆E_Pauli_), orbital (∆E_orb_), and dispersion (∆E_dis_) interaction to the total interaction energy (∆E_int_). Clearly, for X@C_60_ (X = H_2_O, NH_3_), the terms ∆E_elstat_, ∆E_orb_ and ∆E_disp_ act as the stabilizing components, while the ∆E_Pauli_ term constitutes the destabilization factor for X@C_60_. Decomposition of this term indicates that the total attractive interaction between the X and C_60_ is dominated by the ∆E_elstat_ and ∆E_disp_, the covalent term introduced by ∆E_orb_. However, for cationic X^+^@C_60_ (X^+^ = H^+^, H_3_O^+^, NH_4_^+^), the terms ∆E_orb_ and ∆E_disp_ act as the stabilizing components, while the ∆E_Pauli_ and ∆E_elstat_ terms constitute the destabilization factor. In general, the ∆E_elstat_ term is an attractive interaction. The results in Table 2 reinforce the observation that ∆E_elstat_ plays the most significant role in connection with the unfavorable nature of X^+^@C_60_, as the ∆E_Pauli_ is of comparable magnitude to ∆E_disp_, but the electrostatic term has a much more destabilizing effect. The ∆E_int_ results suggest that the interaction between X^+^ and C_60_ is strong but significantly weaker in X@C_60_. The ∆E_orb_ component of ∆E_int_ is rather stronger in X^+^@C_60_ than in X@C_60_, and its magnitude is sufficient to compensate for the destabilizing influence of ∆E_elstat_ and ∆E_Pauli_, which is ultimately responsible for the stability of X^+^@C_60_.

**Figure 3:**
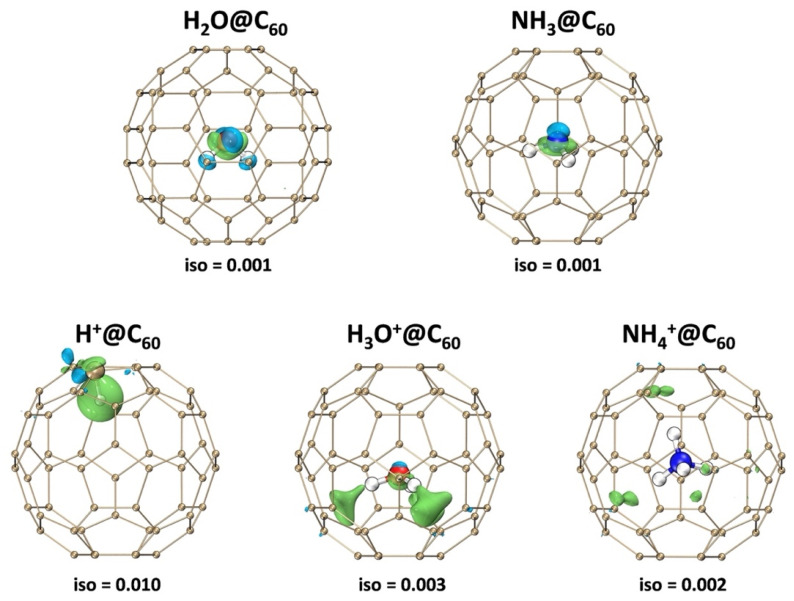
Electron density difference maps of X/X^+^@C_60_ (X^+^ = H^+^, H_3_O^+^, NH_4_^+^, X = H_2_O, NH_3_) and C_60_ cage at the B3LYP-D3(BJ)/6-31G(d, p) level of theory. The green and blue indicate the accumulation and the depletion of the electron density, respectively.

The electron density difference was calculated to explain the orbital interactions between X^+^ and C_60_. Figure 3 displays the electron density difference, with green and blue parts corresponding to regions where the electron density is increased and decreased, respectively. Generally, significant electron polarization between the host and guest leads to a stronger electrostatic interaction. As shown in Figure 3, in X@C_60_, the C_60_ cage has almost no polarization of electron density. This result indicates that the interaction between the carbon cage and X is weak. In the X^+^@C_60_ system, except for H^+^, there is electron accumulation localized in the C_60_ cage, while there is electron depletion on X^+^. This corresponds to the electron transfer resulting from the interaction between X^+^ and C_60_. For the H^+^@C_60_ system, it is noteworthy that the H^+^ shows a very high density of polarized electrons, which verifies the strong binding fore of the C-H bond. Meanwhile, it can be found that there is obvious electron density electron polarization in the H atom of H_3_O^+^@C_60_. The relatively strong electron polarization between the H_3_O^+^ and C_60_ cage suggests the stronger electrostatic interaction between them. However, although the NH_4_^+^ is confined in the C_60_ cage, the cage shows only slight polarization. Our calculation result also indicates that the interaction between NH_4_^+^ and C60 molecules is weak. These findings are thoroughly in accordance with previous studies based on energy decomposition analysis.

In order to quantitatively estimate the interaction between X/X^+^ and C_60_, we performed atomic dipole moment corrected Hirshfeld charge fitting (ADCH), as shown in Table 3. Generally, the ADCH charge is a good charge population analysis method for confined systems [14]. The ADCH charge on X/X^+^@C_60_ is higher than that on free X/X^+^ because the electronegativity of C is greater than that of X/X^+^. Here, it can be seen that the H_2_O (O: −0.714 e) and NH_3_ (N: −0.968 e) possess larger negative charges than H_2_O@C_60_ (O: −0.538 e) and NH_3_@C_60_ (N: −0.538 e), and H_2_O (H: 0.357 e) and NH_3_ (H: 0.323 e) possess larger positive charges than H_2_O@C_60_ (H: 0.266 e) and NH_3_@C_60_ (H: 0.233 e), indicating the interaction between H_2_O, NH_3_ and C_60_. However, the ADCH charges of H^+^@C_60_ (H: 0.053 e) are noticeably smaller than that of free H^+^(H: 1 e), indicating stronger interaction between H^+^ and C_60_. The H_3_O^+^ (O: −0.480 e) has a relatively larger negative charge than H_3_O^+^@C_60_ (O: −0.165 e). The charges are less accumulated in the H_3_O^+^@ C_60_, which implies a charge transfer effect from H_3_O^+^ to the C_60_ cage. In addition, the ADCH charges of H (0.493 e) are the same in the H_3_O^+^, while the ADCH charges of H are not equal in H_3_O^+^@C_60_. This may be because the H_3_O^+^ encapsulated inside the fullerene cage shows a slight deviation from the center. The ADCH charges of NH_4_^+^ (N: −0.249 e) possess a larger negative charge than NH_4_^+^@C_60_ (N: −0.134 e). However, The ADCH charges of NH_4_^+^ (H: 0.312 e) are the same, and the charges of H are also not equal in NH_4_^+^@C_60_.

**Figure 4:**
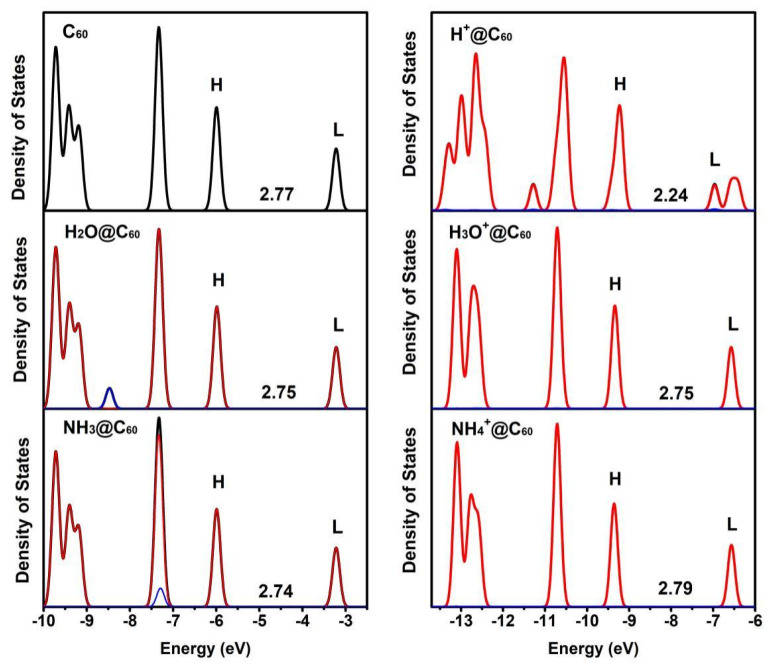
Total density of states (TDOS) and the partial density of states (PDOS) for C_60_, X^+^@C_60_ (X^+^ = H^+^, H_3_O^+^, NH_4_^+^), and X@C_60_ (X = H_2_O, NH_3_) at the B3LYP-D3(BJ)/6-31G(d, p) level of theory. Black represents the TDOS of X/X^+^@C_60_. Red and blue lines represent the contribution of two fragments of the C_60_ cage and X/X^+^ to the DOS, respectively. H and L represent the highest occupied molecular orbital (HOMO) and lowest unoccupied molecular orbital (LUMO), respectively. The energy gap is marked in the figure (unit is kcal/mol).

To gain a deeper insight into the effect of confined species X/X^+^ on the electronic structure of C_60_ cage, the density of states (DOS) of C_60_ and X/X^+^@C_60_ are calculated (as shown in Figure 4). In Figure 4, the red and blue lines represent the contributions of two fragments of C_60_ cage and X/X^+^ to the DOS. H and L represent the highest occupied molecular orbital (HOMO) and lowest unoccupied molecular orbital (HOMO), respectively. The HOMO-LUMO gaps (i.e., the energy difference between HOMO and LUMO levels) of C_60_ and X/X^+^@C_60_ are marked in Figure 4. Compared with the total density of state (TDOS) distributions of C_60_, the TDOS distributions of X@C_60_ are basically unchanged. This implies that there is only a slight interaction between X and C_60_. Moreover, the TDOS is mostly attributed to the contribution from C_60_, indicating that there are few electrons occupied by embedded species in the molecular orbital. The energy gaps of C_60_, H_2_O@C_60_ and NH_3_@C_60_ are 2.77 kcal/mol, 2.75 kcal/mol and 2.74 kcal/mol, respectively. The results reveal that the energy gap of X@C_60_ is slightly smaller than that of the C_60_ cage. Therefore, this indicates a weak interaction between X and the C_60_ cage. This is consistent with the results of previous encapsulated species studies [27,50,51,52]. However, the DOS curves of X^+^@C_60_ change significantly, especially for H^+^@C_60_ and H_3_O^+^@C_60_ in the TDOS curves. This is attributed to the H atom adsorbed on the C_60_ cage in H_3_O^+^@C_60_, which is different from other systems. For the H_3_O^+^@C_60_ system, it indicates a strong interaction between the H_3_O^+^ and C_60_. In contrast, the DOS curves of NH_4_^+^@C_60_ show only slight variations, indicating the weak interaction between NH_4_^+^ and C_60_. Meanwhile, the calculated energy gap of H^+^@C_60_ is 2.24 kcal/mol, which is the lowest energy gap of H^+^@C_60_ among all the systems. This is because H^+^ is attached to the C atom. In the H^+^@C_60_ system, the LUMO orbitals split into two small peaks. This state is a hybrid of C-2p and H-1s orbitals, causing novel peaks in DOS. The energy gaps of H_3_O^+^@C_60_ and NH_4_^+^@C_60_ are 2.75 kcal/mol and 2.79 kcal/mol, respectively. The H_3_O^+^@C_60_ and H_2_O@C_60_ have the same energy gap. In contrast, the energy gap of H_3_O^+^@C_60_ is reduced by 0.02 kcal/mol compared to that of NH_4_^+^@C_60_.

**Figure 5:**
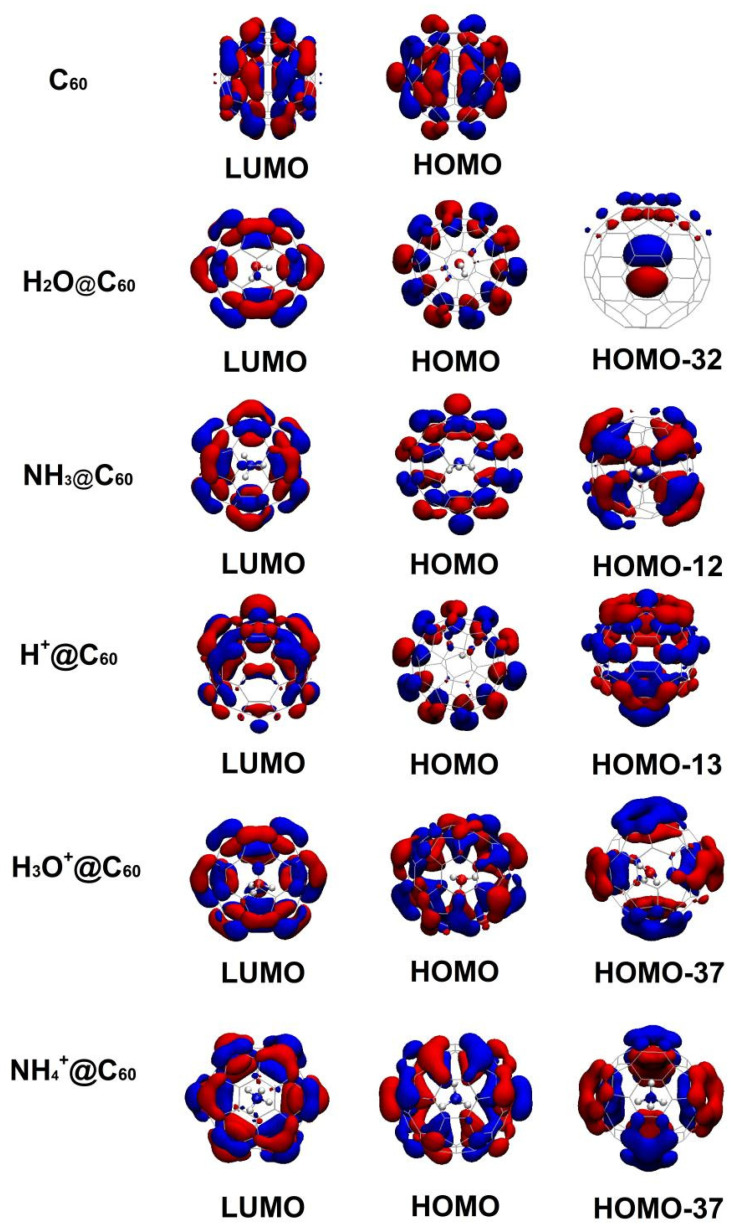
Frontier molecular orbitals (HOMO and LUMO) for C_60_, X^+^@C_60_ (X^+^ = H^+^, H_3_O^+^, NH_4_^+^), and X@C_60_ (X = H_2_O, NH_3_) at the B3LYP-D3(BJ)/6-31G(d, p) level of theory. The molecular orbitals involved for the first time in confined species are also depicted. Molecular orbitals are denoted by blue and red.

To understand the electronic properties, we displayed the frontline molecular orbital diagrams of C_60_ and X/X^+^@C_60_. The HOMO and LUMO orbitals for C_60_ and X/X^+^@C_60_ are plotted in Figure 5. The molecular orbitals involved for the first time by confined species are also depicted in Figure 5. The corresponding frontier molecular orbitals are similar in all these cases. The HOMO iso-surface of C_60_ is symmetrically distributed on its surface, formed by contribution of π-π bonds and some contribution of σ bonds located on C atoms. The LUMO iso-surface is located on C atoms due to contribution of s electrons, and some π-π bonds are displayed on the rest of its surface as well. For X/X^+^@C_60_ systems, the HOMO orbitals are also mainly located on the C_60_ cage, whereas the LUMO orbitals of X/X^+^@C_60_ are located on both confined species and the C_60_ cage, similar to those reported for other endohedral fullerenes [53,54]. For the H_2_O@C_60_ system, the HOMO electronic distributed is on the C_60_ cage, and the π bonds remain in both parts; the LUMO iso-surface is uniformly distributed on the surface with π-π bonds. The HOMO-32 orbital is mainly constructed by the O-2p orbital, with a very small contribution from the C-2p orbital. For the HOMO frontier orbital associated with the NH_3_@C_60_ system, it is concentered on the C_60_ cage displaying π-π bonds, whereas the LUMO electronic distribution is exhibited on C atoms that come from π-π bonds around the surface, alongside the contribution of p electrons. Whereas for the HOMO-12 orbital, the contribution comes from N-2p and C-2p orbitals in NH_3_@C_60_, for the H^+^@C_60_ system, the HOMO iso-surface is concentrated on the C_60_ cage displaying π-π bonds. The electronic distribution of LUMO is located on the C-H bond, whereas π-π bonds are displayed on the surface of the C_60_ cage. It can be seen that the main contribution in HOMO-13 comes from H-1s and C-2p orbitals. For the H_3_O^+^@C_60_ system, the HOMO iso-surface is concentered on the C_60_ cage displaying π-π bonds, the electronic distribution of LUMO is located on the bond of O-H and C_60_ cage, and the HOMO-37 orbital involved in confined species is mainly constructed by the C-2p orbitals and a few H-s orbitals. For NH_4_^+^@C_60_ systems, similar features are shown on the HOMO iso-surface; the electronic distribution of LUMO is located on the C_60_ cage. The HOMO-37 orbital is involved in confined species and is mainly constructed by the C-2p orbital displaying π-π bonds.

**Figure 6:**
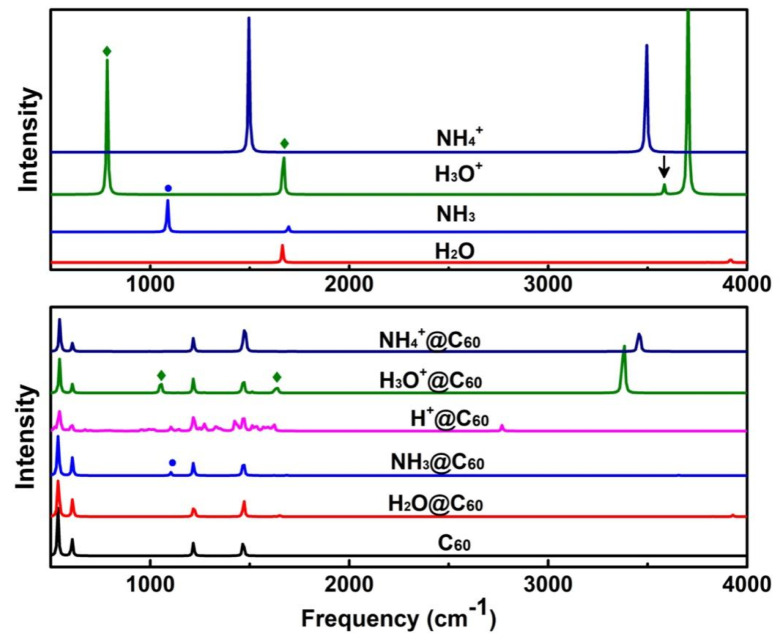
IR spectra for X = H_2_O, NH_3_, X^+^ = H^+^, H_3_O^+^, NH_4_^+^, and X/X^+^@C_60_ at the B3LYP-D3(BJ)/6-31G(d, p) level of theory.

The IR spectra of X = H_2_O, NH_3_, X^+^ = H^+^, H_3_O^+^, NH_4_^+^, and X/X^+^@C_60_ are presented in Figure 6. The vibrational spectra of H_2_O@C_60_, H_3_O^+^@C_60_, and NH_4_^+^@C_60_ share many characteristics with spectrum of C_60_. Figure 6 shows that the spectrum of H_2_O@C_60_ closely resembles the spectrum of the pristine C_60_. In addition, the vibrational features of H_2_O are very weak in the IR spectrum of H_2_O@C_60_, indicating the potential screening effect of the fullerene cage [17]. However, most of the bands in the spectrum of the NH_3_@C_60_ look quite similar to that of C_60_, except for one weak peak observed (marked with a circle in Figure 6). This peak is mainly derived from the N-H wagging mode and is slightly blue-shifted compared to NH_3_. The IR spectrum of H^+^@C_60_ is much more complicated than that of the other embedded systems. This is mainly due to the formation of the C-H bond between the H and C_60_ cage. The IR spectrum of the H_3_O^+^@C_60_ looks quite similar to that of C_60_, except for two weak peaks and a strong peak (marked with a square in Figure 6). These peaks are mainly derived from the O-H stretching mode. For the H_3_O^+^@C_60_ system, except for the O-H free stretch mode, all the normal modes of confined H_3_O^+^ are slightly red-shifted compared to free H_3_O^+^. Interestingly, in the O-H stretching mode, a small peak is observed inside H_3_O^+^ (marked with arrows in Figure 6). The peak change due to encapsulation can be attributed to the interaction effect between H_3_O^+^ and the C_60_ cage. Moreover, the NH_4_^+^@C_60_ and C_60_ spectra show similarity in the whole spectral region, except for the N-H free stretch mode. This result indicates a weak interaction between NH_4_^+^ and the C_60_ cage.

**Figure 7:**
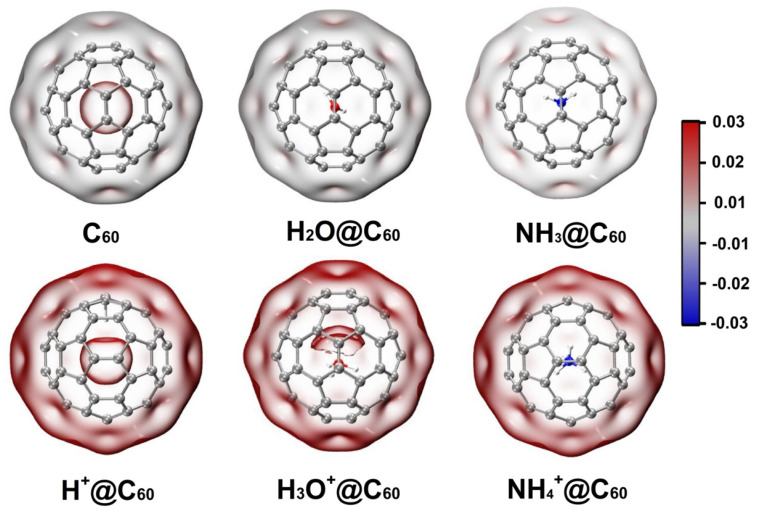
The electrostatic potential of C_60_, X^+^@C_60_ (X^+^ = H^+^, H_3_O^+^, NH_4_^+^), and X@C_60_ (X = H_2_O, NH_3_) at the B3LYP-D3(BJ)/6-31G(d, p) level of theory. Blue and red colors represent negative potential and positive potential, respectively (unit is a.u.).

Electrostatic potential has been widely used in the study of interactions and has become one of the most common means of analyzing the interaction between molecules. Moreover, the electrostatic potential explains the charge distribution on a molecule and indicates electrophilic positively charged or nucleophilic negatively charged properties. Therefore, we calculated the electrostatic potential of C_60_ and X/X^+^@C_60_. Blue and red colors represent negative potential and positive potential, respectively. As shown in Figure 7, for the electrostatic potential of the C_60_ cage, the positive electrostatic potential region is mainly distributed in center of the cage, and the surface of the cage is close to neutral. For the electrostatic potential of X@C_60_, there is no obvious change in the electrostatic potential due to encapsulation of neutral species inside the cage. Encapsulation of X^+^ induces positive electrostatic potential both inside and outside the C_60_ cage; it can be seen from the figure that the electrostatic potential distribution of H^+^@C_60_ in C_60_ cage is different from that of H_3_O^+^@C_60_ and NH_4_^+^@C_60_. This is mainly due to the H^+^ adsorbing on the C atom. For H_3_O^+^@C_60_, the positive electrostatic potential is more distributed on the O atom. This result indicates that there is electron polarization between H_3_O^+^ and C_60_, which is consistent with our previous result. For NH_4_^+^@C_60_, NH_4_^+^ is at the center of the cage, and there is a uniform distribution of the electrostatic potential. However, when the guest species is in an off-centered position, the electrostatic potential is more localized inside the cage. This shows the electron polarization of the X^+^ system compared to the X species located at the center of the cage.

**Figure 8:**
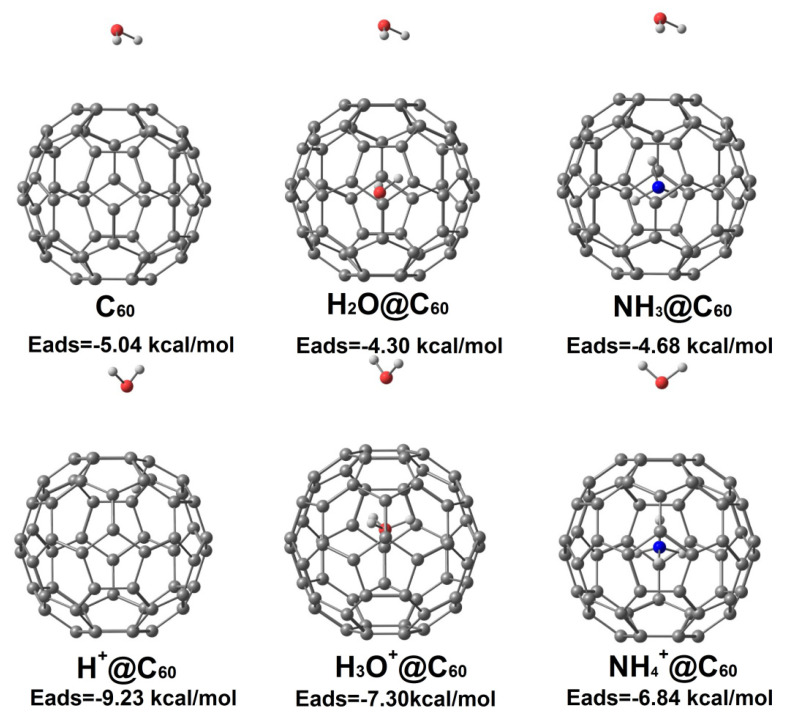
Optimized water adsorption structures on C_60_, X^+^@C_60_ (X^+^ = H^+^, H_3_O^+^, NH_4_^+^), and X@C_60_ (X = H_2_O, NH_3_). In these structures, red, white, cyan, and blue denote O, H, C, and N atoms, respectively.

To explore the effects of H_2_O on the adsorption behavior, we calculated the structure of H_2_O adsorbed on the surface of C_60_, X^+^@C_60_ (X^+^ = H^+^, H_3_O^+^, NH_4_^+^), and X@C_60_ (X = H_2_O, NH_3_). The lowest-energy structures are shown in Figure 8 (for coordinates of the optimized molecular structure, see Supplementary Materials). For H_2_O adsorption on neutral species X@C_60_, the H atoms of H_2_O point towards the C atoms of the C_60_ cage. However, when H_2_O adsorbs on ionic species X^+^@C_60_, the O atom of H_2_O is most likely to point towards the six-membered ring of the C_60_ cage. This result indicates that O atoms prefer to be located in the region of positive electrostatic potential outside the C_60_ cage. For H_2_O adsorbed on H^+^@C_60_, the O atom of H_2_O is close to the C atoms of C_60_. This is probably due to the H atom being absorbed on the C atom and having the lowest adsorption energy (−9.23 kcal/mol). Furthermore, from Figure 8, we can also learn that the interaction between H_2_O and X^+^@C_60_ is much stronger than that with X@C_60_, indicating the presence of positive electrostatic potential.

**Figure 9:**
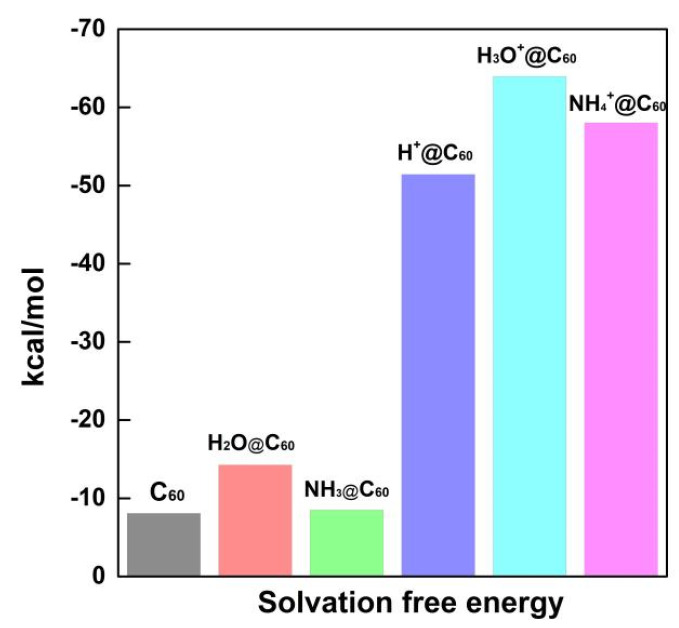
The solvation afree energy of C_60_, X^+^@C_60_ (X^+^ = H^+^, H_3_O^+^, NH_4_^+^), and X@C_60_ (X = H_2_O, NH_3_) (unit is kcal/mol).

To explicitly assess the effect of solvent on X/X^+^@C_60_ interactions, the IEFPCM (integral equation formalism–PCM model) was calculated (see Figure 9). All the negative solvation energies indicate spontaneous solvation. It can be inferred from the above data that a smaller value of solvation energy leads to improved solubility of the complex in the aqueous phase. Solvation studies also reveal that the solvent medium, i.e., water, stabilizes the complex by decreasing its solvation energy. The solvation energies for C_60_, H_2_O@C_60_ and NH_3_@C_60_ are −8.08 kcal/mol, −14.26 kcal/mol, and −8.51 kcal/mol, respectively. It is observed that the solvation energy of C_60_ is slightly higher than the calculated solvation energy of NH_3_@C_60_. Among the neutral species, the solvation energy of H_2_O@C_60_ is the lowest, indicating that H_2_O@C_60_ is stable in the aqueous phase. However, the solvation energies for H^+^@C_60_, H_3_O^+^@C_60_ and NH_4_^+^@C_60_ are −51.42 kcal/mol, −63.94 kcal/mol, and −58.02 kcal/mol, respectively. H_3_O^+^@C_60_ has the lowest solvation energy, followed by NH_4_^+^@C_60_. H^+^@C_60_ has a relatively lower solvation energy, suggesting that H^+^@C_60_ is stable in the aqueous phase. This is consistent with the results from the adsorption energy analysis.

## 4. Materials and Methods

In this work, the cationic X^+^@C_60_ (X^+^ = H^+^, H_3_O^+^, and NH_4_^+^), as well as their corresponding neutral species (X = H_2_O, NH_3_), have been studied using the hybrid generalized gradient approximation (hybrid GGA) B3LYP [55,56] function. Grimme’s empirical dispersion correction with Beck-Johnson damping (D3BJ) was used to consider weak interactions. The 6-31G(d, p) was used to describe all the atoms. In this case, all atoms were allowed to relax. To ensure we obtained the minimum energy structures, vibrational frequency verifications were also performed at the same level of theory. All the structures were assessed with the Gaussian 09 package [57]. The energy decomposition analysis was performed using the B3LYP-D3BJ and TZP basis sets in ADF 2016 [58]. Multiwfn 3.8 [59] programs were used to obtain the electronic structure data.

The interaction energy $∆E_{int}$ can be decomposed into electroatatic interaction $∆E_{elstat}$, repulsive exchange (Pauli) interaction $∆E_{Pauli}$, orbital interaction $∆E_{orb},$ and dispersion interaction $∆E_{disp}$.

(1) $$∆E_{int}=∆E_{elstat}+∆E_{Pauli}+∆E_{orb}+∆E_{disp}$$

where $∆E_{elstat}$ is the conventional electrostatic interaction between two molecules. $∆E_{Pauli}$ is the repulsive Pauli interaction between the occupied orbitals of two different molecules. $∆E_{orb}$ stands for the stabilizing interactions between the occupied molecular orbitals in one molecule and the unoccupied molecular orbitals in the other molecule along with the interaction of occupied and virtual orbitals within the same molecule in the final geometry. $∆E_{disp}$ represents the dispersion forces.

To obtain a deep insight into the nature of the charge transformation, we calculated the electron density difference between X/X^+^ and the C_60_ cage. This can be exactly calculated as

(2) $$∆\rho =\rho_{X/X^{+}@C_{60}}-\rho_{C_{60}}-\rho_{X/X^{+}}$$

where $\rho_{X/X^{+}@C_{60}}$, $\rho_{C_{60}}$, and $\rho_{X/X^{+}}$ represent the electron density of X/X^+^@C_60_, C_60_, and X/X^+^, respectively.

The adsorption energy ($E_{ads}$) is an important factor in measuring the adsorption capacity of a structure. When molecule A is adsorbed on surface B, the calculation formula of adsorption energy is as follows:

(3) $$E_{ads}=E_{H_{2}O-X/X^{+}@C_{60}}-E_{X/X^{+}@C_{60}}-E_{H_{2}O}$$

where $E_{H_{2}O-X/X^{+}@C_{60}}$ is the total energy after H_2_O adsorption, and $E_{X/X^{+}@C_{60}}$ and $E_{H_{2}O}$ are the total energy of adsorbate $X/X^{+}@C_{60}$ and H_2_O, respectively. A negative value indicates a favorable exothermic adsorption.

The integral equation formalism-PCM model (IEFPCM) was applied [60]. Being an essential part of the living system, water was chosen as a solvent with which to study the solubility and stability of the complex. The stability and solubility of X/X^+^@C_60_ can be calculated using the following expression:

(4) $$E_{solvation}=E_{sol}-E_{gas}$$

where $E_{solvation}$ represents system’s total solvation energy, while $E_{sol}$ and $E_{gas}$ stand for the energy in the solvent phase and gas phase, respectively. All the negative solvation energies indicate a spontaneous solvation.

## 5. Conclusions

In this study, we performed a systematic theoretical investigation on the geometric and electronic structures of cationic X^+^@C_60_ (X^+^ = H^+^, H_3_O^+^, and NH_4_^+^) as well as their corresponding neutral species (X = H_2_O, NH_3_). A deeper understanding of cationic X^+^@C_60_ was obtained. Our results revealed that the confined species almost did not change the geometric structure of the C_60_ cage, except for H^+^. For the H^+^@C_60_ system, the H^+^ was attached to C atom in H^+^@C_60_, forming a C-H chemical bond. Meanwhile, we found that orbital interaction plays a significant role between the cationic X^+^ species and the C_60_ cage. Electronic structure analysis showed that the electron polarization of X^+^@C_60_ was stronger than that of X@C_60_. This has important implications for energy applications. For instance, the unique electronic properties of these systems could potentially be harnessed in the development of advanced energy storage materials. The strong electron polarization in X+@C_60_ could lead to more efficient charge transfer and storage capabilities. Additionally, understanding the interactions between protons and the C_60_ cage could contribute to the design of novel fuel cells or batteries that utilize proton conduction. The encapsulated protons could offer a stable and controlled environment for energy conversion processes. We hope that this work will pave the way for further development of proton applications in the future, especially in the field of energy, where innovative solutions are desperately needed to meet the growing demand for sustainable and efficient energy sources.

## Figures and Tables

**Table 1:** Parameters optimized at the B3LYP-D3(BJ)/6-31G(d, p) level of theory (unit is Å).

**Bond Length**	H_2_O	H_2_O@C_60_
r(O-H)	0.965	0.964
r(O-H)	0.965	0.964
	NH_3_	NH_3_@C_60_
r(N-H)	1.018	1.014
r(N-H)	1.018	1.014
r(N-H)	1.018	1.014
		H^+^@C_60_
r(C-H)		1.130
	H_3_O^+^	H_3_O^+^@C_60_
r(O-H)	0.982	0.994
r(O-H)	0.982	0.994
r(O-H)	0.982	0.994
	NH_4_^+^	NH_4_^+^@C_60_
r(N-H)	1.027	1.027
r(N-H)	1.027	1.027
r(N-H)	1.027	1.027
r(N-H)	1.027	1.027

**Table 2:** Energy decomposition analysis of X@C_60_ (X = H_2_O, NH_3_) and X^+^@C_60_ (X^+^ = H^+^, H_3_O^+^, NH_4_^+^) at B3LYP-D3BJ/TZP in kcal/mol.

	∆E_int_	∆E_elstat_	∆E_Pauli_	∆E_orb_	∆E_disp_
H_2_O@C_60_	−13.22	−6.02	13.60	−4.71	−16.09
NH_3_@C_60_	−14.18	−10.60	24.64	−5.95	−22.27
H^+^@C_60_	−174.08	82.43	0.00	−253.88	−2.63
H_3_O^+^@C_60_	−26.50	32.43	19.52	−58.23	−20.22
NH_4_^+^@C_60_	−22.97	33.45	17.07	−47.03	−26.46

**Table 3:** The ADCH charge populations of the neutral species X = H_2_O ad NH_3_ and the cationic species X^+^ = H^+^, H_3_O^+^, NH_4_^+^, and X/X^+^@C_60_ (unit is e).

		ADCH			ADCH
H_2_O	O	−0.714	H_2_O@C_60_	O	−0.538
	H	0.357		H	0.266
	H	0.357		H	0.266
NH_3_	N	−0.968	NH_3_@C_60_	N	−0.680
	H	0.323		H	0.223
	H	0.323		H	0.223
	H	0.323		H	0.222
H^+^	H	1	H^+^@C_60_	H	0.053
H_3_O^+^	O	−0.480	H_3_O^+^@C_60_	O	−0.165
	H	0.493		H	0.268
	H	0.493		H	0.266
	H	0.493		H	0.265
NH_4_^+^	N	−0.249	NH_4_^+^@C_60_	N	−0.134
	H	0.312		H	0.203
	H	0.312		H	0.204
	H	0.312		H	0.206
	H	0.312		H	0.206

## Data Availability

Data is contained within the article or Supplementary Materials.

## Supplementary Materials

The following supporting information can be downloaded at: https://www.mdpi.com/article/10.3390/ijms252212014/s1.
