# Supplementary material for: Encapsulating Proton Inside C60 Fullerene: A Density Functional Theory Study on the Electronic Properties of Cationic X+@C60 (X+ = H+, H3O+ and NH4+)
Source: Int J Mol Sci. 2024 Nov 8;25(22):12014. doi: 10.3390/ijms252212014 (PMC11593435; doi:10.3390/ijms252212014)
Supplement: Supplementary file 1 [file ijms-25-12014-s001.zip › ijms-3288570-supplementary.pdf]

Supplementary Materials for

Encapsulating Proton Inside C_{60} Fullerene: A Density Functional Theory Study on the Electronic Properties of Cationic $X^+@C_{60}$ ($X^+ = H^+$, H_3O^+ and NH_4^+)

Lei Zhao [1], Bo Wang [1,*]

[1] School of Science, Northeast Electric Power University, Jilin, 131200, China; 20172736@neepu.edu.cn
[*] Correspondence: bowang@neepu.edu.cn

Coordinates for all optimized molecular structure

C_{60}

C	0.72745600	-1.00125700	3.32913100
C	1.17704800	0.38244600	3.32913100
C	1.42490000	-1.96120600	2.59579500
C	2.30553600	0.74911400	2.59579500
C	2.30553600	1.98673600	1.83090300
C	0.00000000	1.23762200	3.32913100
C	0.00000000	2.42418400	2.59579500
C	1.17704800	2.80663000	1.83090300
C	-0.69744400	-2.96246300	1.83090300
C	-1.42490000	-1.96120600	2.59579500
C	0.69744400	-2.96246300	1.83090300
C	-0.72745600	-1.00125700	3.32913100
C	-1.17704800	0.38244600	3.32913100
C	-2.60194800	-1.57876000	1.83090300
C	-3.03299200	-0.25214300	1.83090300
C	-2.30553600	0.74911400	2.59579500
C	0.72745600	-3.42544100	-0.59328100
C	-0.72745600	-3.42544100	-0.59328100
C	1.42490000	-3.19882800	0.59328100
C	-1.42490000	-3.19882800	0.59328100
C	-2.60194800	-2.34365200	0.59328100
C	-1.17704800	-2.80663000	-1.83090300
C	-2.30553600	-1.98673600	-1.83090300
C	-3.03299200	-1.75037100	-0.59328100
C	3.03299200	-1.75037100	-0.59328100
C	2.30553600	-1.98673600	-1.83090300
C	2.60194800	-2.34365200	0.59328100
C	1.17704800	-2.80663000	-1.83090300
C	0.00000000	-2.42418400	-2.59579500
C	2.30553600	-0.74911400	-2.59579500
C	1.17704800	-0.38244600	-3.32913100
C	0.00000000	-1.23762200	-3.32913100
C	3.03299200	-0.25214300	1.83090300
C	3.48258500	0.36666800	0.59328100
C	2.60194800	-1.57876000	1.83090300
C	3.48258500	-0.36666800	-0.59328100
C	3.03299200	0.25214300	-1.83090300
C	3.03299200	1.75037100	0.59328100
C	2.60194800	2.34365200	-0.59328100
C	2.60194800	1.57876000	-1.83090300
C	-0.72745600	1.00125700	-3.32913100

C	0.72745600	1.00125700	-3.32913100
C	1.42490000	1.96120600	-2.59579500
C	-1.42490000	1.96120600	-2.59579500
C	-3.03299200	0.25214300	-1.83090300
C	-2.30553600	-0.74911400	-2.59579500
C	-1.17704800	-0.38244600	-3.32913100
C	-2.60194800	1.57876000	-1.83090300
C	-3.03299200	1.75037100	0.59328100
C	-3.48258500	0.36666800	0.59328100
C	-3.48258500	-0.36666800	-0.59328100
C	-2.60194800	2.34365200	-0.59328100
C	-0.72745600	3.42544100	0.59328100
C	-1.17704800	2.80663000	1.83090300
C	-2.30553600	1.98673600	1.83090300
C	-1.42490000	3.19882800	-0.59328100
C	0.69744400	2.96246300	-1.83090300
C	1.42490000	3.19882800	-0.59328100
C	0.72745600	3.42544100	0.59328100
C	-0.69744400	2.96246300	-1.83090300

$H_2O@C_{60}$

C	2.39544100	1.29386300	2.27688800
C	1.38775400	2.34204600	2.27789600
C	3.18196700	1.08361000	1.14530600
C	1.20650600	3.13491000	1.14621200
C	-0.13649600	3.47504200	0.70482300
C	0.23392000	1.85484200	3.01606700
C	-1.05325400	2.18234900	2.59415600
C	-1.24264100	3.01029700	1.41413500
C	2.96244300	-1.36099800	1.41283000
C	2.14290700	-1.14093300	2.59464100
C	3.46962700	-0.27216600	0.70306400
C	1.86465200	0.15903900	3.01547300
C	0.52837400	0.50582900	3.47163200
C	1.09425800	-2.14926100	2.60943300
C	-0.18732500	-1.81595500	3.04723200
C	-0.47583900	-0.46063400	3.48907300
C	2.94118300	-1.38037400	-1.43966400
C	2.40926100	-2.51555100	-0.69973000
C	3.45870000	-0.28189500	-0.75209100
C	2.41984000	-2.50588200	0.69639200
C	1.26464500	-2.99321700	1.43607500
C	1.24283500	-3.01264300	-1.41542800
C	0.13595400	-3.48045700	-0.70560000

C	0.14734500	-3.47060800	0.74973000
C	2.36043000	1.26328800	-2.33043800
C	1.81838200	0.11848800	-3.04511000
C	3.16395200	1.06798400	-1.20807700
C	2.10336800	-1.17596700	-2.61175700
C	1.05415100	-2.18380900	-2.59667900
C	0.47505400	0.45950000	-3.48553300
C	-0.52930800	-0.50684500	-3.47372000
C	-0.23422200	-1.85595200	-3.01843400
C	2.02560200	2.91489000	-0.03492300
C	1.18858000	3.11905000	-1.20625400
C	2.99309700	1.91143900	-0.03553200
C	1.35252700	2.31137700	-2.33015300
C	0.18743400	1.81476700	-3.04413300
C	-0.14757900	3.46567400	-0.74905400
C	-1.26424300	2.99115500	-1.43489200
C	-1.09309800	2.14760300	-2.60662000
C	-2.39751300	-1.29485000	-2.27899100
C	-1.86597900	-0.15999600	-3.01762400
C	-2.14087100	1.13901900	-2.59188700
C	-3.18549100	-1.08411900	-1.14657800
C	-2.02746500	-2.91878100	0.03512800
C	-1.20823000	-3.13971400	-1.14743100
C	-1.38848400	-2.34339000	-2.27874200
C	-2.99540500	-1.91285000	0.03550800
C	-2.36115900	-1.26346100	2.33180200
C	-1.35272100	-2.31252400	2.33128500
C	-1.18979400	-3.12305400	1.20741900
C	-3.16676000	-1.06814300	1.20902500
C	-2.93873300	1.37868100	1.43724700
C	-2.10107100	1.17384500	2.60888800
C	-1.81922700	-0.11927700	3.04780900
C	-3.46324600	0.28197900	0.75216000
C	-2.96049500	1.35966000	-1.41076800
C	-2.41819500	2.50432800	-0.69550800
C	-2.40756000	2.51377700	0.69866600
C	-3.47446000	0.27215800	-0.70349200
O	0.03996100	0.14638100	-0.00180400
H	-0.84376800	-0.23472900	0.05866800
H	0.62415200	-0.61941300	-0.05116100

$NH_3@C_{60}$

C	0.05152800	3.38923900	1.06342200
C	1.43972500	3.17678100	0.67886100

C	-0.46433300	2.75568600	2.19472900
C	2.25469900	2.33897300	1.44229700
C	3.16519000	1.41326600	0.78403600
C	1.50068200	3.12596600	-0.77449700
C	2.37168500	2.23747000	-1.40455500
C	3.22036700	1.36342800	-0.60911100
C	-2.56084900	2.25277600	0.99236300
C	-2.02276200	2.91352200	-0.18740800
C	-1.79848200	2.17516900	2.15836900
C	-0.74393600	3.46962200	-0.15270200
C	0.15146400	3.30694600	-1.28762800
C	-2.46144500	2.17067800	-1.35890300
C	-1.60141800	2.01239400	-2.44497500
C	-0.26828900	2.59215800	-2.40876500
C	-2.51391800	-0.16215300	2.50723200
C	-3.31098600	-0.08126300	1.29170300
C	-1.77374700	0.94213900	2.93077400
C	-3.33344200	1.10106400	0.54947200
C	-3.27181200	1.05085200	-0.90423400
C	-3.22463900	-1.36552300	0.61094300
C	-3.16226200	-1.41219800	-0.78248000
C	-3.18734800	-0.17962100	-1.55594700
C	0.12965200	-0.51799900	3.51181700
C	-0.64159700	-1.66981500	3.06943400
C	-0.42432700	0.76019200	3.44397800
C	-1.93538400	-1.49595100	2.57745800
C	-2.37442700	-2.23948200	1.40598700
C	0.26919700	-2.59523700	2.41237900
C	-0.15131200	-3.30760100	1.28843300
C	-1.50077800	-3.12532500	0.77430600
C	1.71553500	1.67705100	2.62169800
C	2.29344300	0.34286700	2.69212700
C	0.38499600	1.88113300	2.98968300
C	1.51709900	-0.73102800	3.12831300
C	1.60373500	-2.01498500	2.44900900
C	3.18939500	0.18025100	1.55671100
C	3.27104300	-1.04986600	0.90361300
C	2.46212400	-2.17069600	1.35954200
C	-0.05175300	-3.38976300	-1.06323900
C	0.74447000	-3.47137100	0.15233900
C	2.02419400	-2.91491700	0.18701800
C	0.46360500	-2.75260000	-2.19175400
C	-1.71370600	-1.67529600	-2.61944400
C	-2.25086900	-2.33614000	-1.44032000

C	-1.43872400	-3.17600000	-0.67856500
C	-0.38458900	-1.87847900	-2.98661700
C	-0.12925000	0.51742800	-3.50962300
C	-1.51538100	0.73010400	-3.12563000
C	-2.29166700	-0.34269400	-2.69083500
C	0.42415900	-0.75928400	-3.44178200
C	2.51145200	0.16191400	-2.50475000
C	1.93412000	1.49477800	-2.57603600
C	0.64142100	1.66798600	-3.06672800
C	1.77230300	-0.94090200	-2.92831600
C	2.56228500	-2.25314000	-0.99234400
C	3.33266000	-1.10106200	-0.54923000
C	3.30660900	0.08046400	-1.28973500
C	1.79708800	-2.17262700	-2.15554000
N	0.01177600	0.01041800	-0.14487900
H	0.40501100	-0.84852800	0.22363400
H	0.45744900	0.76305900	0.36784500
H	-0.95986600	0.01687700	0.14452600

$H^{+}@C_{60}$

C	-3.37583200	-0.47254800	0.00000000
C	-3.10264900	-1.28220300	1.21288700
C	-2.63559100	-0.58046800	2.32589200
C	-2.54294300	0.88145500	2.29557000
C	-2.94724800	1.64966300	1.18637000
C	-3.42816100	0.97968900	0.00000000
C	-3.10264900	-1.28220300	-1.21288700
C	-2.47171200	-2.49238800	-0.73272300
C	-2.47171200	-2.49238800	0.73272300
C	-1.35235200	-2.96906400	1.42839900
C	-1.48809700	-1.09510100	3.05033800
C	-1.34502900	1.25767500	3.02805200
C	-0.57985000	2.34031100	2.59132700
C	-0.98294100	3.11185700	1.42089600
C	-2.14436700	2.77855800	0.72996600
C	-2.14436700	2.77855800	-0.72996600
C	-2.94724800	1.64966300	-1.18637000
C	-2.54294300	0.88145500	-2.29557000
C	-2.63559100	-0.58046800	-2.32589200
C	-1.35235200	-2.96906400	-1.42839900
C	-0.86104700	-2.26190000	-2.60455600
C	-1.48809700	-1.09510100	-3.05033800
C	-0.69145600	0.04148500	-3.48728400
C	-1.34502900	1.25767500	-3.02805200

C	-0.57985000	2.34031100	-2.59132700
C	-0.98294100	3.11185700	-1.42089600
C	0.22155500	3.48508800	-0.69741500
C	0.22155500	3.48508800	0.69741500
C	-0.69145600	0.04148500	3.48728400
C	3.11752700	1.23813600	1.17631500
C	2.64494100	0.56815800	2.30393500
C	2.55496500	-0.88351500	2.30353500
C	2.94298400	-1.60587900	1.17603400
C	3.43615300	-0.90679300	0.00000000
C	3.11752700	1.23813600	-1.17631500
C	2.46349200	2.45834800	-0.72734700
C	2.46349200	2.45834800	0.72734700
C	1.36706600	2.96113100	1.42540900
C	0.87128600	2.25906300	2.59900400
C	1.49508200	1.08730800	3.02619200
C	1.35336600	-1.26270500	3.03172900
C	0.59199600	-2.34888200	2.60353700
C	0.99692400	-3.10223500	1.42794400
C	2.14437000	-2.73645400	0.72800900
C	2.14437000	-2.73645400	-0.72800900
C	2.94298400	-1.60587900	-1.17603400
C	2.55496500	-0.88351500	-2.30353500
C	2.64494100	0.56815800	-2.30393500
C	1.36706600	2.96113100	-1.42540900
C	0.87128600	2.25906300	-2.59900400
C	1.49508200	1.08730800	-3.02619200
C	0.69817300	-0.04322000	-3.47989400
C	1.35336600	-1.26270500	-3.03172900
C	0.59199600	-2.34888200	-2.60353700
C	0.99692400	-3.10223500	-1.42794400
C	-0.20662900	-3.47798800	-0.69943500
C	-0.20662900	-3.47798800	0.69943500
C	0.69817300	-0.04322000	3.47989400
C	-0.86104700	-2.26190000	2.60455600
C	3.52292100	0.48543600	0.00000000
H	-2.27229400	-0.22947500	0.00000000

$H_3O^+@C_{60}$

C	-0.53325200	-3.15316400	-1.53534500
C	0.23448600	-2.45478800	-2.55704300
C	-1.84304800	-2.76020700	-1.24926300
C	-0.33475300	-1.38627300	-3.25058400
C	0.44750800	-0.19102800	-3.52676000

C	1.61589500	-2.37623600	-2.10691500
C	2.36462300	-1.22851800	-2.36479100
C	1.76876500	-0.11423300	-3.08762800
C	-1.42582000	-3.03036100	1.16271400
C	-0.06186900	-3.44834100	0.86784500
C	-2.30510700	-2.69787500	0.12972800
C	0.37570900	-3.50821200	-0.45536700
C	1.70298000	-3.02565500	-0.80782000
C	0.81069400	-2.90144800	1.89767300
C	2.08018900	-2.43438400	1.55874000
C	2.53622000	-2.49910300	0.17847900
C	-3.15959000	-0.76863500	1.42315700
C	-2.23765500	-1.11446700	2.49830800
C	-3.18932200	-1.54391200	0.26034600
C	-1.39121600	-2.21980400	2.37135300
C	-0.01099400	-2.14361600	2.82937500
C	-1.73644800	0.11996800	3.08733700
C	-0.40937800	0.19558700	3.51757200
C	0.46949800	-0.95859800	3.38799800
C	-3.33135600	0.50276400	-1.12761200
C	-3.30075800	1.30515100	0.08895100
C	-3.27461300	-0.89150400	-1.04036500
C	-3.21771700	0.68542600	1.33904400
C	-2.33961500	1.23262200	2.36855800
C	-2.50116000	2.49331000	-0.18288600
C	-1.65966500	3.01537700	0.80419300
C	-1.57764200	2.37387100	2.10867000
C	-1.69596700	-0.96878100	-2.94570400
C	-1.75907800	0.48282200	-3.04021700
C	-2.43613400	-1.64052900	-1.96831000
C	-2.55859100	1.20224800	-2.14916800
C	-2.04810900	2.43469500	-1.56617100
C	-0.43062600	0.96344500	-3.39556600
C	0.05325800	2.14602500	-2.83565100
C	-0.77198000	2.89582400	-1.90002600
C	0.57205800	3.15807900	1.53966500
C	-0.33302000	3.50448900	0.45408300
C	0.10156800	3.44302300	-0.87069700
C	1.87594100	2.75630000	1.25089200
C	1.74053800	0.97484700	2.95423200
C	0.37751300	1.39054300	3.25136100
C	-0.19507700	2.45873200	2.56084400
C	2.47288100	1.64303000	1.97363100
C	3.34942600	-0.50050300	1.12101400

C	2.58303600	-1.19871200	2.14142200
C	1.79730600	-0.47685000	3.03935000
C	3.29477800	0.88983800	1.03872800
C	3.17853200	0.76929300	-1.42276500
C	3.23603200	-0.68172200	-1.33639600
C	3.31990600	-1.30338900	-0.09185900
C	3.20754100	1.53812600	-0.26064500
C	1.46429100	3.03022700	-1.16994800
C	1.43425600	2.22806400	-2.38357200
C	2.27237000	1.12041700	-2.50530100
C	2.33038300	2.69116800	-0.13062300
O	-0.45143900	-0.01251100	0.02758400
H	-0.80059000	0.91728000	0.05646000
H	-0.83288100	-0.47310300	-0.76636500
H	-0.74674200	-0.49085600	0.84767800

$NH_4^+@C_{60}$

C	0.59444600	-2.43329600	-2.52202000
C	-0.85203100	-2.28345600	-2.58838800
C	1.18461300	-3.03823900	-1.41193100
C	-1.64877300	-2.74732900	-1.54062700
C	-2.77279300	-1.94762900	-1.07273400
C	-1.14513300	-1.00130100	-3.21289400
C	-2.22219400	-0.23562800	-2.76516600
C	-3.05313900	-0.71858800	-1.67177500
C	2.98031500	-1.34178000	-1.39872400
C	2.36244500	-0.70851600	-2.55586900
C	2.40219300	-2.48175800	-0.83891200
C	1.19541800	-1.24249800	-3.10566400
C	0.12073200	-0.35790800	-3.53354700
C	2.50491600	0.73384900	-2.40978500
C	1.47415800	1.58161600	-2.82021400
C	0.25714000	1.02384300	-3.39339500
C	2.82456800	-1.61408000	1.43317000
C	3.42808200	-0.42396600	0.84946600
C	2.32259600	-2.62085600	0.60767400
C	3.50392300	-0.29059800	-0.53759900
C	3.21027700	0.99193600	-1.16187100
C	3.05497900	0.71979900	1.67013000
C	2.77313800	1.94851700	1.07167000
C	2.85229200	2.08720700	-0.37503700
C	0.34264000	-2.87420900	2.06373700
C	0.86437000	-1.82248700	2.92480000
C	1.05597800	-3.26322500	0.92928400

C	2.07830200	-1.20585400	2.61526300
C	2.22054200	0.23641700	2.76176100
C	-0.25969300	-1.02389700	3.39365500
C	-0.12349900	0.35804600	3.53389900
C	1.14265100	1.00142900	3.21144000
C	-1.03320900	-3.38006900	-0.38170800
C	-1.77737500	-2.97205200	0.80229600
C	0.35383900	-3.52217500	-0.31915000
C	-1.10412800	-2.72433300	1.99940000
C	-1.47665000	-1.58142500	2.82075000
C	-2.85232200	-2.08664600	0.37533200
C	-3.20917900	-0.99106900	1.16312800
C	-2.50639000	-0.73334900	2.41177000
C	-0.59598300	2.43238100	2.52265100
C	-1.19818700	1.24274700	3.10694300
C	-2.36427800	0.70827300	2.55815200
C	-1.18559300	3.03883900	1.41270500
C	1.03363100	3.37883500	0.38118200
C	1.64800000	2.74583600	1.53912000
C	0.85072600	2.28375900	2.58688400
C	-0.35345200	3.52264500	0.31919900
C	-0.34397400	2.87302300	-2.06442600
C	1.10292800	2.72492700	-1.99888100
C	1.77699100	2.97131600	-0.80231300
C	-1.05691300	3.26405900	-0.92996400
C	-2.82366600	1.61281600	-1.43505500
C	-2.07986400	1.20595900	-2.61827300
C	-0.86684400	1.82278000	-2.92616800
C	-2.32325300	2.61986800	-0.60845300
C	-2.97986400	1.34046700	1.40028500
C	-3.50236400	0.29059500	0.53753700
C	-3.42605400	0.42371100	-0.84964600
C	-2.40293900	2.48095200	0.83950800
N	0.01242100	0.00089700	0.00404100
H	0.55405400	-0.14711700	0.86450700
H	0.64395600	-0.01264000	-0.80624200
H	-0.46206700	0.91052900	0.05123700
H	-0.68544700	-0.74644900	-0.09341000

H_2O-C_{60}

C	0.32240900	-0.20712300	3.54201200
C	1.68918100	0.16193100	3.20593500
C	-0.15838800	-1.47461900	3.21418100
C	2.51792500	-0.75187900	2.55482100

C	3.38456500	-0.30660200	1.47431800
C	1.69089000	1.56016500	2.80376800
C	2.52163800	1.98714300	1.76808000
C	3.38658000	1.03409700	1.08933500
C	-2.31038900	-0.50469500	2.49387300
C	-1.80912800	0.81744500	2.83569200
C	-1.50207200	-1.62613100	2.67824500
C	-0.52013700	0.96299700	3.34874100
C	0.32562100	2.05506900	2.89204900
C	-2.30723300	1.75792100	1.84381900
C	-1.49595000	2.80439800	1.40573600
C	-0.15219700	2.95688100	1.94127100
C	-2.24400100	-2.55567800	0.51527500
C	-3.08696900	-1.38571400	0.32082100
C	-1.46836100	-2.67275000	1.66836600
C	-3.11996100	-0.38220300	1.29117400
C	-3.11840300	1.01828700	0.88901700
C	-3.05147500	-1.03390400	-1.09099900
C	-3.04942700	0.30583500	-1.47561800
C	-3.08340700	1.35348200	-0.46595400
C	0.42995100	-3.52476100	0.34208000
C	-0.37884900	-3.40199400	-0.86118900
C	-0.10340600	-3.16920700	1.58068100
C	-1.68768900	-2.92765700	-0.77671600
C	-2.18621900	-1.98636400	-1.76824100
C	0.48806300	-2.95689300	-1.94166100
C	0.01010100	-2.05501400	-2.89233800
C	-1.35501800	-1.55989800	-2.80388100
C	2.01602800	-2.07399100	2.21258500
C	2.57302100	-2.44605700	0.92094900
C	0.70626400	-2.42813600	2.53566100
C	1.79670100	-3.15647500	0.00529000
C	1.83215300	-2.80470200	-1.40619000
C	3.41878500	-1.35367600	0.46451500
C	3.45318800	-1.01687000	-0.88873500
C	2.64338200	-1.75788900	-1.84392300
C	0.01328700	0.20721800	-3.54224200
C	0.85608900	-0.96290900	-3.34917500
C	2.14492300	-0.81732300	-2.83594000
C	0.49417400	1.47452900	-3.21435000
C	-1.68022700	2.07361000	-2.21296200
C	-2.18234200	0.75136700	-2.55452000
C	-1.35321200	-0.16189200	-3.20585800
C	-0.37061100	2.42755000	-2.53565200

C	-0.09406800	3.52538300	-0.34236600
C	-1.46049100	3.15564700	-0.00571800
C	-2.23686500	2.44580900	-0.92139800
C	0.43923700	3.16838500	-1.58045000
C	2.57989100	2.55561700	-0.51575200
C	2.02331900	2.92780400	0.77604700
C	0.71453300	3.40243700	0.86069600
C	1.80437200	2.67318600	-1.66899800
C	2.64651000	0.50487200	-2.49390600
C	3.45472500	0.38159300	-1.29029000
C	3.42231300	1.38534800	-0.32225200
C	1.83815500	1.62625600	-2.67864600
O	-6.11584000	-0.02408100	-0.08544500
H	-5.76227200	0.83147000	0.18783900
H	-5.73991000	-0.63435700	0.56103000

H_2O-H_2O@C_{60}

C	0.31585600	-2.60399700	2.41156200
C	1.68139500	-2.10351700	2.42624400
C	-0.16352400	-3.29032500	1.29543400
C	2.50951100	-2.30940900	1.32384100
C	3.37650600	-1.24056100	0.85478200
C	1.68196300	-0.81731900	3.10458200
C	2.51184200	0.20778900	2.65359300
C	3.37808700	-0.00849900	1.50599000
C	-2.31831300	-2.09267000	1.44617900
C	-1.81781800	-1.37541900	2.60937600
C	-1.50673500	-3.02780700	0.80221400
C	-0.52807400	-1.62567400	3.08153100
C	0.31681300	-0.52124600	3.51068800
C	-2.31696700	-0.00934700	2.54586800
C	-1.50538600	1.04908900	2.95680100
C	-0.16181500	0.78798200	3.45050700
C	-2.24444300	-2.19924100	-1.40146600
C	-3.08887600	-1.22199200	-0.73222700
C	-1.47046900	-3.08290800	-0.65102600
C	-3.12703500	-1.17104700	0.66261900
C	-3.12637800	0.11814300	1.34278500
C	-3.05211500	0.00981900	-1.50599300
C	-3.05436000	1.24277800	-0.85589800
C	-3.09063600	1.29898300	0.59782000
C	0.42896300	-2.77662900	-2.19239600
C	-0.37827400	-1.85437700	-2.97490000
C	-0.10550400	-3.38016600	-1.05573400

C	-1.68736900	-1.57247700	-2.58938600
C	-2.18541800	-0.20783900	-2.65264000
C	0.48866300	-0.78600100	-3.44517800
C	0.01048200	0.52175400	-3.50928200
C	-1.35437100	0.81663900	-3.10304400
C	2.01074600	-3.02662000	0.16107500
C	2.56881500	-2.39969300	-1.02633600
C	0.70234800	-3.50763000	0.14664300
C	1.79465500	-2.27807700	-2.17865300
C	1.83135900	-1.04750300	-2.95239200
C	3.41351500	-1.29675900	-0.59755700
C	3.44900400	-0.11754300	-1.33919800
C	2.64110700	0.00956500	-2.54139800
C	0.01210600	2.60295100	-2.41020600
C	0.85538800	1.62521800	-3.08095600
C	2.14206700	1.37392300	-2.60588600
C	0.49212800	3.29179300	-1.29531500
C	-1.68616100	3.02912700	-0.16098900
C	-2.18725500	2.31222500	-1.32457300
C	-1.35443900	2.10303600	-2.42441300
C	-0.37517600	3.50813200	-0.14596100
C	-0.10172800	2.78020000	2.19741100
C	-1.46851900	2.28030000	2.18150000
C	-2.24415500	2.40215100	1.02803100
C	0.43372900	3.38044500	1.05770900
C	2.57147400	2.19902800	1.40162300
C	2.01297800	1.57229900	2.58938300
C	0.70542800	1.85772100	2.98087300
C	1.79956800	3.08569700	0.65187000
C	2.64169900	2.09030300	-1.44295800
C	3.44872900	1.16808900	-0.65993900
C	3.41511300	1.22201800	0.73231800
C	1.83570700	3.03068000	-0.80159100
O	0.26445300	-0.06706900	-0.08889100
H	-0.00158800	0.85606800	-0.17553400
H	-0.29688100	-0.40793200	0.61762400
O	-6.12033900	0.03950000	-0.08854800
H	-5.76902300	0.46721600	0.70216300
H	-5.74399400	-0.84829600	-0.04489500

H_2O-NH_3@C_{60}

C	3.12160900	-1.07824800	0.81458700
C	3.12343900	0.28936500	1.31955300
C	3.09052800	-1.31465400	-0.56159000

C	3.09272000	1.36193600	0.42486100
C	2.24715200	2.51348600	0.70303500
C	2.31221300	0.32258200	2.52677800
C	1.50171700	1.42579800	2.79023600
C	1.46811400	2.54240900	1.85948100
C	1.46667400	-3.14636500	-0.23500000
C	1.50085000	-2.90025900	1.19874900
C	2.24398500	-2.37116400	-1.09611900
C	2.30910500	-1.88575100	1.71180400
C	1.81073900	-1.02083700	2.76920800
C	0.15671800	-3.09243900	1.72105900
C	-0.32228400	-2.25893900	2.73142600
C	0.52129700	-1.20277900	3.26677900
C	0.37874900	-2.23596600	-2.70677100
C	-0.43191900	-3.04644200	-1.81001100
C	1.68802400	-1.90534500	-2.35732400
C	0.10058200	-3.49298600	-0.59953700
C	-0.70936700	-3.45909600	0.60993800
C	-1.79819600	-2.54685200	-1.86415200
C	-2.57374700	-2.51263600	-0.70407100
C	-2.01855700	-2.97868000	0.55857600
C	1.36094400	0.39680600	-3.18489400
C	-0.00559500	0.05203500	-3.54790100
C	2.19033100	-0.56154500	-2.60177900
C	-0.48610700	-1.23593000	-3.31401400
C	-1.83134100	-1.42774200	-2.79372200
C	-0.84917400	1.20461000	-3.27082200
C	-2.13931800	1.02150200	-2.77146300
C	-2.64086300	-0.32301300	-2.52752900
C	3.05775700	1.11343100	-1.01007600
C	2.19162900	2.11288900	-1.61686600
C	3.05704300	-0.19538800	-1.49240800
C	1.36157800	1.76201300	-2.68228800
C	-0.00424400	2.26143600	-2.73541900
C	1.69144700	2.97845900	-0.55895000
C	0.38213600	3.45641500	-0.60953800
C	-0.48360400	3.09142500	-1.72127700
C	-3.45063000	1.07728900	-0.81570800
C	-2.64040300	1.88751400	-1.71379700
C	-1.82942200	2.90070800	-1.19920800
C	-3.41331600	1.31333400	0.55875300
C	-3.38142800	-1.11231800	1.00759400
C	-3.41648400	-1.35929700	-0.42562900
C	-3.45152500	-0.28834000	-1.31918700

C	-3.37959000	0.19597900	1.48969300
C	-1.68685200	-0.39578400	3.18215100
C	-1.68717600	-1.75966500	2.67836000
C	-2.51783400	-2.11233500	1.61581600
C	-2.51508200	0.56187700	2.60024000
C	-0.70583400	2.23424000	2.70449800
C	0.15810500	1.23525300	3.31230800
C	-0.32155800	-0.05157600	3.54514100
C	-2.01432000	1.90501600	2.35632200
C	-1.79413500	3.14772700	0.23469700
C	-0.42819400	3.49109600	0.59864800
C	0.10358900	3.04288800	1.80716500
C	-2.56895400	2.36982800	1.09474000
N	-0.17983600	0.02571700	0.14550400
H	-0.71355700	0.63266200	-0.46701200
H	0.79269900	0.17156200	-0.10180700
H	-0.39846200	-0.92102800	-0.14444400
O	6.11308300	0.02700000	-0.08946900
H	5.76161300	-0.84615700	0.12433800
H	5.76156700	0.58512500	0.61527700

H_2O-H^+@C_{60}

C	-3.05704100	-0.68814200	-0.84661900
C	-3.06824300	-1.44941200	0.42327900
C	-2.95052400	-0.69549000	1.59133900
C	-2.93576700	0.76817600	1.52800500
C	-3.07952500	1.48052700	0.32279800
C	-3.19506700	0.75058300	-0.91898200
C	-2.43825400	-1.50801200	-1.91783700
C	-1.88586000	-2.66336100	-1.24434700
C	-2.26648900	-2.62774400	0.17115700
C	-1.34115000	-3.01542000	1.14939900
C	-1.99999600	-1.11664000	2.60423800
C	-1.98925300	1.23909000	2.52682100
C	-1.19967500	2.35865300	2.25968600
C	-1.32855300	3.07432800	0.99568000
C	-2.24958400	2.64696100	0.04379200
C	-1.87078600	2.61310000	-1.36650900
C	-2.46312900	1.42431900	-1.96897300
C	-1.74237900	0.65929100	-2.90608400
C	-1.74129600	-0.80672600	-2.90276800
C	-0.59969900	-3.08298900	-1.60924300
C	0.13870900	-2.37472200	-2.64693300
C	-0.41710700	-1.26175800	-3.28454500

C	0.39990300	-0.08604100	-3.54526800
C	-0.41845200	1.09537200	-3.31836500
C	0.14436600	2.23596200	-2.74305700
C	-0.59157000	3.00679400	-1.74788000
C	0.36139500	3.47523000	-0.75407400
C	-0.00040500	3.50820900	0.59222000
C	-1.40882000	0.07926000	3.18656000
C	2.79491800	1.46662500	1.88928000
C	2.08504300	0.79431500	2.88356900
C	2.08054800	-0.66014300	2.91748600
C	2.78838400	-1.38181500	1.95740800
C	3.52931600	-0.68011800	0.92069000
C	3.40479100	1.41063300	-0.38216900
C	2.58911300	2.59624200	-0.16539400
C	2.21156000	2.63041100	1.23905000
C	0.94444400	3.07660800	1.61073200
C	0.20212800	2.37202800	2.64382500
C	0.75961400	1.25408400	3.26382500
C	0.75470700	-1.09932000	3.32568300
C	0.19326800	-2.24234600	2.75882100
C	0.93155900	-2.99508600	1.75812200
C	2.19864600	-2.57137600	1.36353800
C	2.57679600	-2.60637700	-0.04195300
C	3.39879600	-1.43749600	-0.31425900
C	3.27638000	-0.76944200	-1.53181700
C	3.28050200	0.68499400	-1.56623500
C	1.68405400	3.00965200	-1.14147000
C	1.55000600	2.24876000	-2.37361200
C	2.32944200	1.11079900	-2.57971000
C	1.74261000	-0.07983100	-3.17728500
C	2.32788100	-1.24326500	-2.52871900
C	1.54406100	-2.36610300	-2.26758800
C	1.67238700	-3.06311600	-0.99812600
C	0.34464800	-3.49968500	-0.58996500
C	-0.01842200	-3.46594100	0.76013900
C	-0.06246100	0.08506900	3.54101300
C	-1.21304400	-2.25055900	2.38284600
C	3.53363000	0.71418300	0.88776700
H	-2.00249800	-0.37691400	-0.57660200
O	-5.82683700	-0.01685500	-0.02843100
H	-6.46648200	-0.73762200	-0.08983100
H	-6.36263900	0.75868900	0.18000800

H_2O-H_3O^+@C_{60}

C	-0.45581000	-0.93167200	3.36656400
C	0.38796500	-2.02773100	2.90919200
C	-1.75511700	-0.79171400	2.87280900
C	-0.09943400	-2.94013600	1.97242200
C	0.75066000	-3.39793900	0.88377100
C	1.75094100	-1.53067800	2.79885400
C	2.56442500	-1.96385400	1.75207100
C	2.05433600	-2.91496500	0.77543100
C	-1.45559200	1.64601400	2.69146400
C	-0.10165400	1.50453200	3.21031200
C	-2.27234100	0.52491100	2.52722400
C	0.38812700	0.24121800	3.54136300
C	1.75099100	-0.12878800	3.18876500
C	0.75188800	2.45390800	2.51019500
C	2.05604400	2.09782200	2.16772800
C	2.56587200	0.78002300	2.51454000
C	-3.08868300	1.38153700	0.35575600
C	-2.22945400	2.54701700	0.52453200
C	-3.10643700	0.38805200	1.33767200
C	-1.43358400	2.67914900	1.66683200
C	-0.07129800	3.18197700	1.55668700
C	-1.69423300	2.91572200	-0.77956100
C	-0.38472700	3.39051900	-0.88475600
C	0.44176800	3.52847300	0.30641100
C	-3.09230500	-1.36952000	-0.40610000
C	-3.07405600	-0.32764000	-1.42582600
C	-3.10706500	-1.01561000	0.94602500
C	-3.07534800	1.02032800	-1.05669600
C	-2.21272000	1.96744200	-1.75556100
C	-2.20411500	-0.77812100	-2.50479500
C	-1.38101700	0.13159700	-3.17620500
C	-1.38608700	1.53584900	-2.79581000
C	-1.45098000	-2.78961900	1.45011500
C	-1.43981900	-3.16207700	0.04337800
C	-2.26327600	-1.73925500	1.88934100
C	-2.24061400	-2.46323700	-0.86320200
C	-1.69566900	-2.10024900	-2.16327100
C	-0.07540900	-3.53395100	-0.30771900
C	0.44095500	-3.18488600	-1.55542100
C	-0.38649200	-2.45126100	-2.50187300
C	0.82145500	0.93783800	-3.37056200
C	-0.01893000	-0.23826500	-3.53622300
C	0.46785600	-1.50304200	-3.20394300
C	2.11511900	0.79719300	-2.86912000

C	1.82196300	2.79899500	-1.45596200
C	0.46973400	2.94254700	-1.97480200
C	-0.02139800	2.03340800	-2.91191200
C	2.62688400	1.74787900	-1.89379300
C	3.43501200	1.36521400	0.40601600
C	2.59300800	2.45947600	0.86400000
C	1.80489600	3.16224200	-0.04707500
C	3.45102500	1.01680500	-0.94358800
C	3.43309400	-1.37922400	-0.35587200
C	3.41725700	-1.01574600	1.05254400
C	3.41774700	0.32720500	1.42561200
C	3.44945200	-0.38506200	-1.33217800
C	1.82088000	-1.64910800	-2.68891500
C	1.80407300	-2.68787100	-1.66995700
C	2.59270500	-2.55417800	-0.52747500
C	2.62479900	-0.52154500	-2.52263800
O	-0.29469900	-0.01703900	-0.01336500
H	-0.61210400	-0.20637100	-0.93638000
H	-0.66824200	-0.70657900	0.59847800
H	-0.63908000	0.87222500	0.26417600
O	-5.93199600	0.00077900	0.00461100
H	-6.52707200	0.75303800	0.11429100
H	-6.52353000	-0.74994800	-0.13140700

H_2O-NH_4^+@C_{60}

C	-2.06227700	2.45378800	1.73557100
C	-2.60617700	2.55425100	0.38900200
C	-0.75928200	2.87947700	1.99764100
C	-1.82291500	3.07759100	-0.64090800
C	-1.83643100	2.45058700	-1.95565900
C	-3.43549700	1.38212700	0.14830400
C	-3.44860300	0.78176200	-1.11112600
C	-2.63216000	1.32712500	-2.18555600
C	-0.36664600	0.90693400	3.43190400
C	-1.72575600	0.46179900	3.15605400
C	0.10627100	2.09059900	2.86342500
C	-2.55610300	1.21854500	2.32686300
C	-3.40527500	0.55616600	1.34684800
C	-1.70989800	-0.99041500	3.04060200
C	-2.52443700	-1.62477200	2.10079800
C	-3.39004300	-0.83479300	1.23630800
C	2.27669600	1.02042300	2.37239300
C	1.78326600	-0.21457300	2.96672300
C	1.45676100	2.14895700	2.32318300

C	0.48923800	-0.27072600	3.48495800
C	-0.34058200	-1.44283000	3.24356700
C	2.30263600	-1.32829900	2.18510900
C	1.50755500	-2.45222000	1.95567200
C	0.15699000	-2.51036700	2.49550900
C	2.21803000	2.63560300	0.02476700
C	3.07597500	1.46064200	0.07643800
C	1.42643300	2.97319700	1.12373100
C	3.10259400	0.66946900	1.22581300
C	3.11924800	-0.78204100	1.11035900
C	3.06372200	0.83491700	-1.23900000
C	3.07852500	-0.55565000	-1.34934600
C	3.10737500	-1.38123900	-0.15021700
C	-0.46323800	3.52269700	-0.36813300
C	0.36377600	3.17169800	-1.51496200
C	0.05736300	3.42560000	0.92330700
C	1.67600800	2.73735300	-1.32255600
C	2.19619800	1.62293000	-2.10242300
C	-0.48491800	2.50915600	-2.49625500
C	0.01402900	1.44152700	-3.24402400
C	1.38378600	0.98994000	-3.04427500
C	1.73362300	-2.45136400	-1.73628800
C	2.22834200	-1.21648100	-2.32846400
C	1.39940300	-0.46087800	-3.15901600
C	0.43127800	-2.87893200	-1.99810500
C	0.13515000	-3.52205000	0.36873900
C	1.49423500	-3.07727700	0.64114600
C	2.27615500	-2.55240900	-0.38892800
C	-0.38550600	-3.42605300	-0.92276100
C	-2.54755900	-2.63692600	-0.02486000
C	-2.00459200	-2.73953500	1.32179500
C	-0.69183100	-3.17201100	1.51479900
C	-1.75570400	-2.97376700	-1.12380200
C	-2.60672200	-1.01978200	-2.37345900
C	-3.43232400	-0.66945600	-1.22664000
C	-3.40398100	-1.46089500	-0.07802900
C	-1.78556800	-2.14741500	-2.32327800
C	0.04024500	-0.90540200	-3.43116200
C	-0.81682500	0.27031300	-3.48410300
C	-2.11164800	0.21429500	-2.96650100
C	-0.43411100	-2.08919100	-2.86384300
N	-0.13763900	0.00188100	0.01524700
H	0.86798100	-0.00430700	0.22865900
H	-0.67578000	-0.03983200	0.88926700

H	-0.36719700	-0.81174600	-0.56735200
H	-0.37413700	0.86387500	-0.49002700
O	5.94004400	0.00067800	-0.00093600
H	6.51200800	-0.76556600	0.13164500
H	6.55445300	0.72868300	-0.15731500
